# Supplementary material for: Frequency-Dependent Ecological Interactions Increase the Prevalence, and Shape the Distribution, of Preexisting Drug Resistance
Source: PRX Life. Author manuscript; Available in PMC 2025 Aug 8. (PMC12333505; doi:10.1103/prxlife.2.023010)
Supplement: supps [file NIHMS2045714-supplement-supps.pdf]

Supplemental Material: Frequency-dependent ecological interactions increase the prevalence, and shape the distribution, of pre-existing drug resistance

Simulations

Here we provide additional complimentary results from our numerical simulations in the main paper.

Additional generalized Moran process results

Similar to the main text figure, we compare the extinction time distributions of two emerging mutants in an initially ancestor population. Here we compare a neutral mutant with a negative mutant.

While the effect is smaller than the comparison between positive and neutral mutants in the main text, we find similar qualitative trends in that extinction time distribution for neutral mutants is shifted to longer extinction times when compared to the negative mutant (**Fig.** S1).

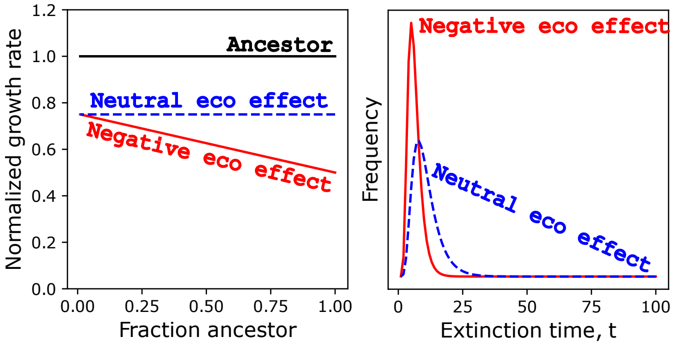

Figure S1. Similar qualitative trends exist when comparing neutral and negative mutants. Closed form extinction time distributions are calculated and visualized for a generalized Moran process (N=100, $f_c = 0.25$). The red distribution results from a mutant with a negative ecological interaction with the ancestor ($f_e = 0.5$), while the blue population has no ecological interaction with the ancestor ($f_e = 1 - fc = 0.75$).

Visualization of sample evolutionary trajectories on a log-axis.

Similar to the main text figure, we visualize representative evolutionary trajectories under the rare and many mutant regimes. This time, the y-axis is on a log scale, which better highlights the presence of low-frequency deleterious mutants in the trajectories (**Fig.** S2).

Mutant-mutant game assay control experiment

Each of the three engineered cell lines (BRAF, KRAS, PIK3CA) and the evolved gefitinib-resistant cell line exhibited a positive ecological interaction when co-cultured with the gefitinib-sensitive ancestor PC9 cell line. Our analytical approximations and simulations suggest this is because of the strong selective advantage the positive ecological interactions convey to the resistant mutants. That is, if the pool of drug-resistant mutants contains mutants with strong positive ecological interactions, they will be selected for. However, this does not suggest that these mutants will exhibit ecological interactions with one another, as the selective pressure to confer such an interaction is missing. We hypothesized that the engineered BRAF and KRAS mutants would not exhibit the same positive ecological interaction we observed between each mutant and their ancestor. This hypothesis turned out to be right in this instance (**Fig.** S3). In addition, this experiment serves as a technical control experiment for the game assay, as it reinforces the observed ecological interactions are not a technical artifact (such as one that may arise from a finite error rate in counting of fluorescent cells).

Analytical theory

The following sections describe the analytical theory supporting the numerical simulations in the main text. Important formulas that are used to fit the simulation results are highlighted by boxes.

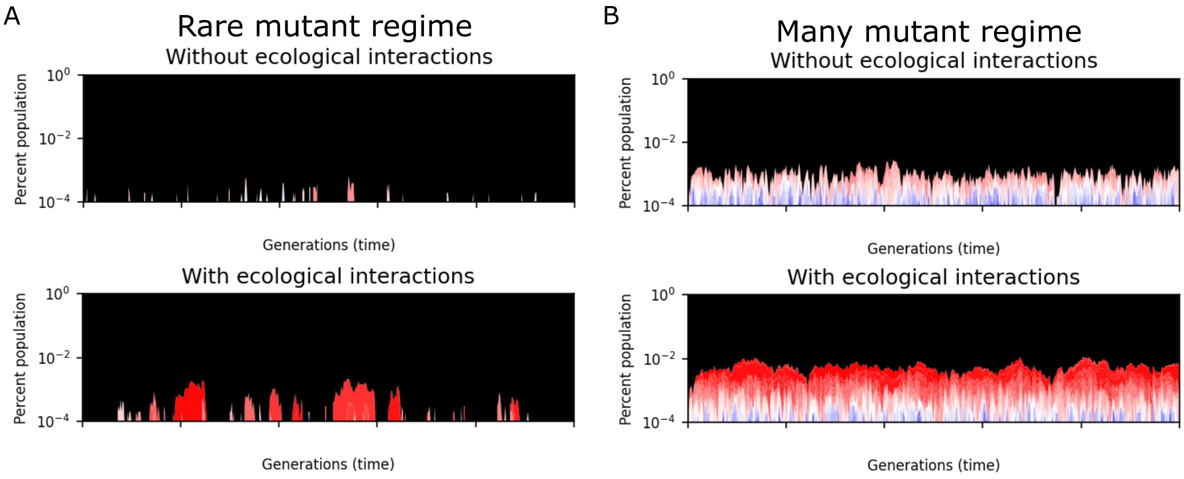

Figure S2. Positive ecological interactions make pre-existence more likely and dominate the stationary distribution of mutants (log-scale). (A) Representative Wright-Fisher trajectory in the "rare mutant regime". Black corresponds to the ancestral population. Mutants exist in higher fractions and for longer periods with ecological interactions. Each mutant is colored by its ecological fitness where red represents an f_e value near 1 and blue represents an f_e value near 0. (B) Representative trajectory in the "many mutant regime". Strong positive ecological interactions dominate the stationary distribution of mutants (visually the mutants appear red, not blue).

Stationary distribution and total number of mutants

We seek to derive an approximate analytical expression for the stationary probability density of mutants $P(f)$ in the Wright-Fisher simulations described in the main text. We assume ecological interactions are present, and that sufficient time has passed for a stationary state to be reached. $P(f)df$ is defined as the fraction of the total population that consists of mutants with instantaneous fitnesses between f and $f+df$. Integrating this distribution gives the mean total number of mutants $N_{\text{mut}}^{\text{eco}} = N\int_0^{f_{\max}} df\, P(f)$, where N is the fixed total population size. For the theoretical calculations we consider an upper bound on the mutant fitness $f_{\max} < 1$ to allow for a well-defined normalization (as explained in more detail below), though we can set $f_{\max}$ arbitrarily close to 1 in order to fit the numerical results.

Every generation of the model consists of a mutation step followed by selection. Let us consider first the mutation part. If $P(f)$ is the current distribution, mutation modifies it to a new distribution,

$$P^m(f) = P(f) + \mu\rho_\alpha(f) - \mu P(f). \tag{S1}$$

Here μ is the mutation probability for a single cell in one generation, and $\rho_\alpha(f)$ is the probability density of a new mutant having fitness f, given a total fraction of mutants $\alpha \equiv \int_0^{f_{\max}} df\, P(f) = N_{\text{mut}}^{\text{eco}}/N$. The second and third terms on the right-hand side in **Eq.** (S1) are respectively the gain and loss due to new mutations.

The dependence of $\rho_\alpha(f)$ on α reflects the role of ecological interactions. In the simplest linear model, a new mutant is assigned a fitness function $f(\alpha) = \alpha f_i + (1-\alpha) f_e$, where f_i (the intrinsic fitness) is randomly drawn from a uniform distribution between 0 and $1-f_c$, and f_e (the ecological fitness) is randomly drawn from a uniform distribution between 0 and $f_{\max}$. Here f_c, where $0 < f_c < 1$, is the cost associated with intrinsic fitnesses, with $1-f_c < f_{\max}$. For this definition of $f(\alpha)$, the distribution $\rho_\alpha(f)$ is given by

$$\rho_\alpha(f) = \begin{cases} \frac{f}{\alpha(1-\alpha)f_{\max}(1-f_c)} & f < \alpha(1-f_c) \\ \frac{1}{(1-\alpha)f_{\max}} & \alpha(1-f_c) \le f < (1-\alpha)f_{\max} \\ \frac{\alpha(1-f_c)+(1-\alpha)f_{\max}-f}{\alpha(1-\alpha)f_{\max}(1-f_c)} & f \ge (1-\alpha)f_{\max} \end{cases} . \tag{S2}$$

For $0 < \alpha < 1$ the distribution $\rho_\alpha(f)$ has a trapezoidal shape, rising linearly from zero for small f, then plateauing in the middle region, before decreasing linearly to zero at $f_{\max}$. In the two limits $\alpha = 0$ and $\alpha = 1$ it reverts to a uniform distribution between 0 and $f_{\max}$ (for $\alpha = 0$) or between 0 and $1-f_c$ (for $\alpha = 1$).

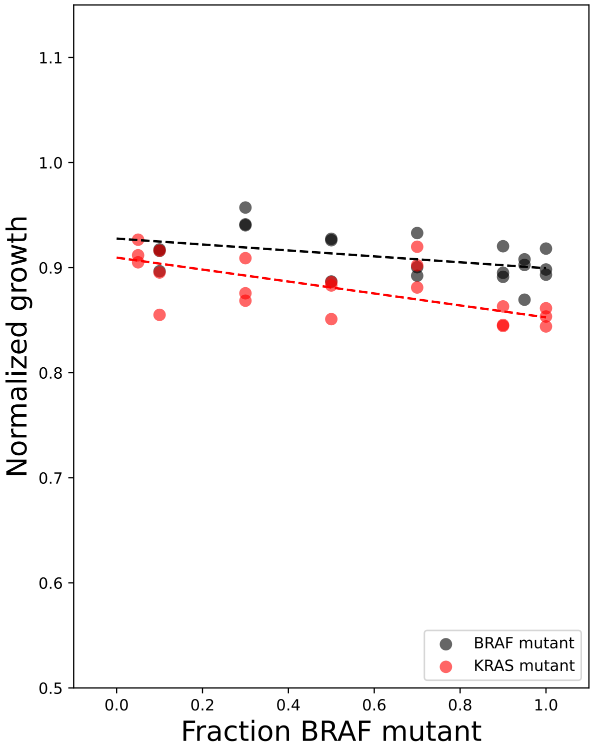

Figure S3. Engineered BRAF and KRAS cell lines do not exhibit a positive ecological interaction when co-cultured. Frequency-dependent growth rate measurements between an engineered BRAF cell line (black) and an engineered KRAS cell line (red).

The second part of the dynamics is the selection step, which makes a further modification of the mutant distribution, yielding

$$P^s(f) = \frac{f P^m(f)}{1 - \int_0^{f_{\max}} df\, P^m(f) + \int_0^{f_{\max}} df\, f P^m(f)}. \tag{S3}$$

The above form reflects the fact that the ancestors after the mutation step, comprising a fraction $1 - \int_0^{f_{\max}} df\, P^m(f)$ of the total, have fitness 1, and mutants with fitness f have a chance of surviving into the next generation proportional to f. In order for the system to be in a stationary state, the distribution $P^s(f)$ in the next generation must end up being the same as the starting distribution $P(f)$ in the current generation. Using **Eqs.** (S1)-(S3), we can express the condition $P^s(f) = P(f)$ compactly as

$$P(f) = \frac{f\,[(1-\mu)P(f) + \mu\rho_\alpha(f)]}{1-\beta}, \tag{S4}$$

where $\beta \equiv \int_0^{f_{\max}} df\,(1-f)\,[(1-\mu)P(f) + \mu\rho_\alpha(f)]$). **Eq.** (S4) can be solved for $P(f)$,

$$P(f) = \frac{f\mu\rho_\alpha(f)}{1-\beta-f(1-\mu)}. \tag{S5}$$

Note that both α and β on the right-hand side of **Eq.** (S5) depend implicitly on $P(f)$. In order for the solution to be self-consistent, we plug **Eq.** (S5) into the definitions of α and β, which leads to a closed system of equations for these two quantities:

$$\alpha = \int_0^{\mathrm{fmax}} df\, \frac{f\mu\rho_\alpha(f)}{1-\beta-f(1-\mu)}, \qquad \beta = \int_0^{\mathrm{fmax}} df\, \frac{(1-\beta)(1-f)\mu\rho_\alpha(f)}{1-\beta-f(1-\mu)}. \tag{S6}$$

The equation for β is satisfied exactly when $\beta = \mu$, using the fact that $\int_0^{f_{\max}} df\, \rho_\alpha(f) = 1$. Plugging $\beta = \mu$ into the α equation, we can carry out the integral analytically, leading to the following relation:

$$
\begin{aligned}
\alpha = \frac{\mu}{\alpha(\alpha-1)(1-\mu)(1-f_c)\,f_{\max}} \Bigg[& \alpha f_c \Big(\ln\big[\alpha(f_c-1)+1\big] \\
& - \ln\big[\alpha(f_c+f_{\max}-1) - f_{\max}+1\big] + (\alpha-1) f_{\max} \Big) - (\alpha-1)\Big(\ln\big[\alpha(f_c-1)+1\big] \\
& + (f_{\max}-1)\ln\big[\alpha(f_c+f_{\max}-1)-f_{\max}+1\big] + \alpha f_{\max} \Big) \\
& + ((\alpha-1)f_{\max}+1)\ln\big[(\alpha-1)f_{\max}+1\big] \Bigg].
\end{aligned}
\tag{S7}
$$

There is no explicit analytical solution for α from the above equation, but there are ways to derive approximate solutions that work in different limits. We consider two such limits in turn.

Small mutant fractions ($\mu \ll 1$): When $\mu \ll 1$, the mean number of mutants in the population becomes small, and their fraction α, given by the solution of **Eq.** (S7), scales like $\alpha \propto \mu$. In this limit **Eq.** (S7) gives us $\alpha = \mu(-f_{\max}^{-1}\ln(1-f_{\max}) - 1) + \mathscr{O}(\mu^2)$, so we get an approximate expression for the total mutant fraction (main text **Eq.** (3)):

$$
\boxed{N_{\text{mut}}^{\text{eco}} \approx N\mu\left(-\frac{\ln(1-f_{\max})}{f_{\max}} - 1\right) \qquad \text{for } \mu \ll 1.}
\tag{S8}
$$

To leading order in μ, we can approximate the stationary distribution of mutant fitnesses in **Eq.** (S5) as (main text Eq. (5)):

$$
\boxed{P(f) \approx \frac{f\mu}{f_{\max}(1-f)} \qquad \text{for } \mu \ll 1.}
\tag{S9}
$$

Note that since $\alpha \ll 1$, $\rho_\alpha(f) \approx \rho_0(f)$, and the distribution of instantaneous fitnesses f in **Eq.** (S9) is approximately also the distribution of ecological fitnesses f_e. In order for **Eq.** (S9) to be normalizable, and hence its integral giving **Eq.** (S8) to be well-defined, we need $f_{\max}$ strictly smaller than 1. In the numerical simulations only a finite number of mutants are sampled overall during the course of the evolutionary trajectories, and an effective value of $f_{\max} \approx 0.995$ was found to provide good fits between the theory and simulation results.

Large mutant fractions ($\mu \lesssim 1$): We would like to extend the results above to larger values of μ and α, to cover simulation cases where the mutant fractions α are on the order of 10% of the total. We note that as α gets larger, the distribution of new mutant fitnesses $\rho_\alpha(f)$ in **Eq.** (S2) gets increasingly suppressed at fitnesses near zero and $f_{\max}$. This makes the precise value of $f_{\max}$ less important for determining α, and we can approximate **Eq.** (S7) by taking the $f_{\max} \to 1$ limit. If we then keep the expressions to leading order in μ, assuming that $\alpha \sim \mathscr{O}(\mu)$, **Eq.** (S7) becomes

$$
\alpha \approx \frac{\mu}{1-f_c}\big((f_c-1)\ln(\alpha) + f_c \ln f_c\big).
\tag{S10}
$$

The solution to this equation has the form

$$
\alpha \approx \mu W\left(\left(\mu f_c^{f_c/(f_c-1)}\right)^{-1}\right).
\tag{S11}
$$

Here $W(x)$ is the Lambert W function, which is the solution y of the equation $ye^y = x$. For $x > 0$ (which is the only case that arises in our problem) the function is single-valued (the so-called zero branch of the solution).

While **Eq.** (S11) works in the $f_{\max} \to 1$ limit, ideally we would like an expression that works for $f_{\max}$ close to, but not exactly 1, and for the entire range of $\mu \lesssim 1$ including small μ. Since we know $N_{\text{mut}}^{\text{eco}} = N\alpha$ for $\mu \ll 1$ and $f_{\max} < 1$ from **Eq.** (S8), we posit the following approximate form for $N_{\text{mut}}^{\text{eco}}$ (main text Eq. (4)):

$$
\boxed{N_{\text{mut}}^{\text{eco}} \approx \frac{N\mu}{f_{\max}} W\left(\left(1 - f_{\max} + \mu f_c^{f_c/(f_c-1)}\right)^{-1}\right).}
\tag{S12}
$$

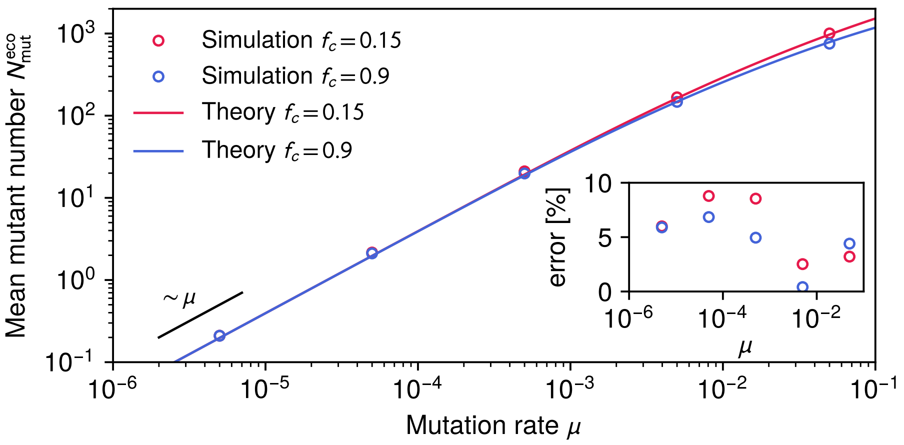

Figure S4. Comparison of the approximate analytical theory, **Eq.** (S12) (curves), and the simulation results (circles) for the mean number of mutants $N_{\text{mut}}^{\text{eco}}$ as a function of mutation rate μ. We set $f_{\max} = 0.995$, $N = 10^4$, and use two different values for the fitness cost, $f_c = 0.15$ and 0.9 (red and blue respectively). The inset shows the absolute percent error of the analytical approximation with respect to the numerical values. The black line on the left shows the scaling $N_{\text{mut}}^{\text{eco}} \propto \mu$ expected in the small μ regime from **Eq.** (S8).

By construction this is consistent with **Eq.** (S11) when $f_{\max} \to 1$. When $1 - f_{\max}$ is small but nonzero and $\mu \ll 1$, we can use the fact that $W(x)$ diverges like $\ln x$ for large positive x to see that **Eq.** (S12) gives $N_{\text{mut}}^{\text{eco}} \approx -N\mu f_{\max}^{-1} \ln(1 - f_{\max})$ for $\mu \to 0$. This recovers the dominant contribution in Eq. (S8) when $1 - f_{\max}$ is small.

Thus in principle **Eq.** (S12) should work for a wide range of μ and different values of f_c. **Fig. S4** depicts a comparison of **Eq.** (S12) to simulation results, and the analytical approximation is within 10% of the numerical value across five decades of μ and both small and large f_c (as shown by the errors shown in the inset). For small μ the $N_{\text{mut}}^{\text{eco}}$ curves start out independent of f_c and proportional to μ, as expected from **Eq.** (S8). With increasing μ, the curves bend downwards in a way that depends on f_c, as the population of mutants becomes a non-negligible fraction of the total.

Non-uniform ecological/intrinsic fitness distributions: The derivations above can be easily extended to non-uniform distributions of the ecological and/or intrinsic fitnesses. This modifies the shape of $\rho_\alpha(f)$, depending on the specific distributions from which f_i and f_e are drawn for new mutants. In general, imagine f_e is drawn from a distribution $\rho_0(f_e)$, like the Gaussian example considered in the main text. In the limit $\mu \ll 1$, when the total mutant fraction α is small, **Eq.** (S9) for the distribution becomes (main text Eq. (6)):

$$\boxed{P(f_e) \approx \frac{f_e \mu}{(1 - f_e)} \rho_0(f_e) \qquad \text{for } \mu \ll 1.} \tag{S13}$$

Other results from the theory can be similarly generalized.

No ecological interactions: In the absence of ecological interactions, the fitness function $f(\alpha) = f_i$ for all α. If f_i is drawn from a uniform distribution between 0 and $1 - f_c$, the distribution $\rho_\alpha(f)$ becomes uniform: $\rho_\alpha(f) = 1/(1 - f_c)$. An analogous calculation to the one above yields the mean number of mutants in this scenario (main text Eq. (2)):

$$\boxed{N_{\text{mut}}^{\text{no eco}} = N\mu \left(-\frac{\ln f_c}{1 - f_c} - 1 \right).} \tag{S14}$$

This number is a useful baseline for gauging the relative effectiveness of ecological interactions in enhancing mutant populations.

Mean time to extinction of an individual mutant

The final quantity we would like to calculate theoretically is the mean number of generations that an individual mutant survives after first arising. Note that if a mutant is generated by the mutation step, but does not survive the selection step immediately afterwards, we say its lifetime is zero generations. To simplify the calculation, we consider the regime $\mu \ll 1$, where the chance that a mutant disappears via a second mutation is negligible. And by the definition of the model, the same type of mutant cannot

be generated again from either the ancestor or other mutant populations. So in this regime the mutant persists with a randomly fluctuating population until in one of the selection steps none of its population is chosen to survive to the next generation.

Let us focus on a single mutant type with fitness f. If there are ℓ such mutants in the current generation, the probability $W_{k\ell}$ that there will be k mutants of this type in the next generation is given by the binomial distribution characteristic of Wright-Fisher dynamics,

$$W_{k\ell} = \binom{N}{k} \left(1 - \frac{f\ell}{N(1-\beta)}\right)^{N-k} \left(\frac{f\ell}{N(1-\beta)}\right)^{k}. \tag{S15}$$

For $\mu \ll 1$ we can assume that $\beta \ll 1$, $f \approx f_e$ and that $N \gg k$ for any k that has a non-negligible probability, since the number of mutants of a single type at any given time will be a tiny fraction of the total. We can then approximate **Eq.** (S15) as

$$W_{k\ell} \approx \frac{(f_e\ell)^k}{k!} e^{-f_e\ell}, \tag{S16}$$

which is just the limit in which the binomial distribution looks like a Poisson distribution. The probabilities $W_{k\ell}$ can be interpreted as components of an $N \times N$ transition matrix W. Since the vast majority of this matrix will consist of probabilities exponentially close to zero, we can focus on the states $k, \ell = 0, \ldots, M$ for some $M \ll N$. Thus we will consider W instead to be an $(M+1) \times (M+1)$ matrix, choosing M large enough to get a satisfactory approximation to the non-truncated system. This reduces the problem to an $(M+1)$ state discrete time Markov process.

Note that $W_{k0} = 0$, so $\ell = 0$ (extinction) is an absorbing state. From the theory of phase-type distributions[61], the mean number of generations to extinction can be calculated using the $M \times M$ submatrix S of the tranpose W^T, defined via $S_{k\ell} = W_{\ell k}$ for $k, \ell = 1, \ldots, M$. Starting from a population of 1 (after the mutation step) at generation zero, the mean time to extinction is given by

$$\tau(f_e) = \mathbf{z}^T (I - S)^{-1} \mathbf{e} - 1. \tag{S17}$$

Here I is the $M \times M$ identity matrix, $\mathbf{z}$ is an M-dimensional vector with a 1 in the first element and zero elsewhere, and $\mathbf{e}$ is an M-dimensional vector with 1 for all its elements. The -1 at the end of **Eq.** (S17) is due to the counting convention where extinction during the first selection step is considered to be an extinction time of zero.

For a given choice of M, it turns out the matrix inverse in **Eq.** (S17) can be calculated analytically. Even though the resulting expression becomes unwieldy for large M, it can always be Taylor expanded around $f_e = 0$ to give relatively simple results. The Taylor coefficient of order f_e^n in the expansion remains unchanged for any choice of $M \geq n$. Thus we can find the Taylor expansion of $\tau(f_e)$ in the non-truncated system up to any chosen order M, simply by Taylor expanding **Eq.** (S17).

The first few terms of this Taylor expansion are:

$$\tau(f_e) = f_e + \frac{f_e^2}{2} + \frac{2f_e^3}{3} + \frac{f_e^4}{8} + \frac{19f_e^5}{30} + \frac{f_e^6}{144} + \frac{107f_e^7}{280} + \cdots. \tag{S18}$$

While the lowest terms are sufficient to describe the mean extinction time of mutants with $f_e \ll 1$, progressively more terms are required to approximate $\tau(f_e)$ as f_e approaches 1 from below. In fact, technically in this approximation the series $\tau(f_e)$ diverges at $f_e = 1$, since we have effectively taken $N \to \infty$ in **Eq.** (S16). In practice this is not a problem since we only consider mutants with fitnesses up to $f_{\max} < 1$. Rather than working with the Taylor expansion directly, we constructed an analytical approximation designed to agree with the expansion through order f_e^3, and capture the divergence at large f_e (main text Eq. (1)):

$$\boxed{\tau(f_e) \approx \frac{3\ln(1-f_e)}{f_e^2 - 3}.} \tag{S19}$$

Despite its simple form, this approximation agrees with the simulation results for $\mu \ll 1$ across the whole range of f_e with a typical error of 5%.
